# Hearing Dysfunction in a Large Family Affected by Dominant Optic Atrophy (OPA8-Related DOA): A Human Model of Hidden Auditory Neuropathy

**DOI:** 10.3389/fnins.2019.00501

**Published:** 2019-05-28

**Authors:** Rosamaria Santarelli, Chiara La Morgia, Maria Lucia Valentino, Piero Barboni, Anna Monteleone, Pietro Scimemi, Valerio Carelli

**Affiliations:** ^1^Department of Neurosciences, University of Padova, Padua, Italy; ^2^Audiology Service, Santi Giovanni e Paolo Hospital, Venice, Italy; ^3^Audiology and Phoniatrics Service, Treviso Regional Hospital, Treviso, Italy; ^4^Dipartimento di Scienze Biomediche e Neuromotorie, Università di Bologna, Bologna, Italy; ^5^IRCCS Istituto delle Scienze Neurologiche di Bologna, UOC Clinica Neurologica di Bologna, Bologna, Italy; ^6^Studio Oculistico D’Azeglio, Bologna, Italy

**Keywords:** hearing impairment, electrocochleography, speech perception, optic atrophy, acoustic reflexes

## Abstract

Hidden auditory neuropathy is characterized by reduced performances in challenging auditory tasks with the preservation of hearing thresholds, resulting from diffuse loss of cochlear inner hair cell (IHC) synapses following primary degeneration of unmyelinated terminals of auditory fibers. We report the audiological and electrophysiological findings collected from 10 members (4 males, 6 females) of a large Italian family affected by dominant optic atrophy, associated with the *OPA8* locus, who complained of difficulties in understanding speech in the presence of noise. The patients were pooled into two groups, one consisting of 4 young adults (19–50 years) with normal hearing thresholds, and the other made up of 6 patients of an older age (55–72 years) showing mild hearing loss. Speech perception scores were normal in the first group and decreased in the second. Otoacoustic emissions (OAEs) and cochlear microphonics (CMs) recordings were consistent with preservation of outer hair cell (OHC) function in all patients, whereas auditory brainstem responses (ABRs) showed attenuated amplitudes in the first group and severe abnormalities in the second. Middle ear acoustic reflexes had delayed peak latencies in all patients in comparison with normally hearing individuals. Transtympanic electrocochleography (ECochG) recordings in response to 0.1 ms clicks at intensities from 120 to 60 dB peak equivalent SPL showed a reduction in amplitude of both summating potential (SP) and compound action potential (CAP) together with delayed CAP peak latencies and prolonged CAP duration in all patients in comparison with a control group of 20 normally hearing individuals. These findings indicate that underlying the hearing impairment in OPA8 patients is hidden AN resulting from diffuse loss of IHCs synapses. At an early stage the functional alterations only consist of abnormalities of ABR and ECochG potentials with increased latencies of acoustic reflexes, whereas reduction in speech perception scores become apparent with progression of the disease. Central mechanisms increasing the cortical gain are likely to compensate for the reduction of cochlear input.

## Introduction

Dominant optic atrophy (DOA) is amongst the most common inherited optic neuropathies, with a prevalence of about 1 in 25,000–30,000 (Lenaers et al., 2012; Yu-Wai-Man and Chinnery, 2013), and is characterized by a slowly progressive bilateral visual loss beginning in childhood (Kjer, 1959). Typically, there is selective neurodegeneration of retinal ganglion cells (RGCs) mostly affecting the small fibers of the papillo-macular bundle that leads to temporal pallor at fundus examination, central scotoma and loss of visual acuity (Carelli et al., 2004; Yu-Wai-Man et al., 2011). Up to 75% of DOA cases are due to dominant heterozygous mutations affecting the *OPA1* gene (Alexander et al., 2000; Delettre et al., 2000; Lenaers et al., 2012). Missense mutations in the GTPase domain result in a multisystemic disease involving central and peripheral nervous systems, skeletal muscle and stability of mitochondrial DNA (Amati-Bonneau et al., 2008; Hudson et al., 2008; Yu-Wai-Man et al., 2010). Besides optic atrophy, sensorineural hearing loss is the earliest and most frequent clinical feature associated with *OPA1* missense mutations (Yu-Wai-Man et al., 2010; Leruez et al., 2013).

The genetic basis of DOA not linked to *OPA1* mutations is increasingly being elucidated, and a growing list of other genes involved in the recurrent association of syndromic DOA and sensorineural deafness is being reported. This includes *OPA3* with the peculiar association with cataracts (Reynier et al., 2004; Sergouniotis et al., 2015), Wolframin 1 (Eiberg et al., 2006; Rendtorff et al., 2011), *MFN2* (Rouzier et al., 2012), *SPG7* (Klebe et al., 2012), and *DNM1L* (Gerber et al., 2017). Almost all these genes encode proteins involved in mitochondrial functions, affecting mitochondrial dynamics and oxidative phosphorylation (Yu-Wai-Man et al., 2016).

In previous studies we characterized sensorineural deafness in patients harboring missense mutations in the *OPA1* gene (Huang et al., 2009; Santarelli et al., 2015). Our findings indicate that underlying the hearing impairment is a disordered synchrony of auditory nerve fiber activity resulting from neural degeneration of their distal portion (Santarelli et al., 2015). Indeed, OPA1 patients displayed the clinical profile of auditory neuropathy (AN) consisting of moderate hearing loss with disproportionate impairment of speech perception, abnormal brainstem responses and presence of normal otoacoustic emissions (OAE) (Starr et al., 1996). Transtympanic electrocochleography (ECochG) showed normal receptor potentials, cochlear microphonic (CM) and summating potential (SP), consistent with preservation of hair cell activity, whereas the synchronous compound action potential (CAP) recorded in normally hearing individuals was replaced by a prolonged negative low-amplitude response, which is believed to originate from the activity of degenerated neural terminals based on the effects of adaptation induced by high stimulation rates.

The term “hidden auditory neuropathy” was coined recently (Schaette and McAlpine, 2011) to indicate the auditory dysfunction resulting from diffuse loss of inner hair cell (IHCs) synapses in the cochlea with preservation of hearing thresholds. The decrease in number of synaptic contacts is consequent to primary degeneration of unmyelinated neural terminals (see Liberman and Kujawa, 2017 for a review). Studies performed in animal models revealed that behavioral hearing thresholds are relatively unaffected until IHC loss exceeds 40–60% (Liberman and Kujawa, 2017; Salvi et al., 2017). In both humans and in animal models, the reduction of the cochlear output results in a decrease in amplitude of ABR Wave I and in reduced performance in challenging auditory tasks relying on temporal cues such as speech understanding in noisy environments. Hidden AN is believed to result from acoustic trauma (Liberman and Kujawa, 2017), aging (Parthasarathy and Kujawa, 2018), and ototoxic drug administration (Liberman and Kujawa, 2017; Salvi et al., 2017).

In a previous paper we described a new form of DOA in a large family of Italian descent whose affected subjects complained of difficulties in understanding speech and showed an increased central conduction time at somato-sensory evoked potentials recording, together with cardiac abnormalities (OPA8) (Carelli et al., 2011). Genome-wide linkage revealed a locus on chromosome 16 in the critical interval 16q21–q22. In this study we present the specific features of the hearing dysfunction affecting this family. The findings collected from audiological studies, speech perception evaluation and transtympanic electrocochleography are consistent with hidden AN.

## Materials and Methods

### Subjects

We evaluated hearing function in 10 subjects (4 males; mean age 52.6 years; age range 19–71) from the original family mapping at the *OPA8* locus. Clinical details from all patients are summarized in Table 1, whereas audiological results are reported in Table 2.

**Table 1:** Clinical and neurological findings from OPA8 patients.

Case	Skeletal muscle biopsy	mtDNA deletion	Other Clinical features
			
#1	Normal	None	Optic atrophy, prolapse mitral valve, ↑central conduction at SSEPs
#2	n.a.	–	Optic atrophy
#3	n.a.	–	Optic atrophy
#4	n.a.	–	Optic atrophy
#5	Slight myopathic changes with diffuse increase of subsarcolemmal SDH staining and a few COX negative fibers	Low amount (Age related)	Optic atrophy, prolapse mitral valve, ↑central conduction at SSEPs
#4	n.a.	–	Optic atrophy
#7	n.a.	–	Optic atrophy
#9	n.a.	–	Optic atrophy
#2	Normal	None	Optic atrophy, mitral insufficiency, ↑central conduction at SSEPs
#10	Parcellar increase of subsarcolemmal SDH staining	Low amount (Age related)	Optic atrophy, prolapse mitral valve, ↑central conduction at SSEPs


**n.a., not available; COX, cytochrome c oxidase; SDH, succinate dehydrogenase; SSEPs, somatosensory evoked potentialsxf*.*

**Table 2:** Audiological Data from OPA8 subjects.

Subjects#	#1	#2	#3	#4	#5	#6	#7	#8	#9	#10
**Clinical**										
Gender	F	F	M	F	F	M	M	F	F	M
Age tested	48	27	19	50	72	55	65	68	69	64
Age onset	22	9	10	20	26	9	11	18	18	17
Sign at onset	Vision	Vision	Vision	Hearing	Vision	Vision	Vision	Vision	Vision	Vision
Deafness	Noise	Noise	Noise	Noise	Quiet	Quiet	Quiet	Quiet	Noise	Noise
Deaf onset	-	24	-	8	64	40	-	-	-	54
Tinnitus	-	-	-	-	Yes	-	Yes	-	Yes	Yes
Vertigo	-	-	-	-	-	-	-	-	Yes	-
**Audiology**	R/L									
Hearing	N/N	N/N	N/N	N/N	Mild/Mild	Mild/Mild	Mild/Mild	Mild/Mild	Mild/N	Mild/Mild
PTA (dB)	10/10	10/11	11/15	11/11	29/34	20/25	33/31	31/34	30/10	21/21
LF (dB)	10/10	10/11	13/16	10/11	26/25	16/15	23/23	24/26	23/10	18/15
HF (dB)	10/15	20/13	10/10	13/22	43/60	32/58	55/53	53/52	53/17	38/42
Stapedial Reflexes^∗^	+/+	+/+	+/+	+/+	+/+	+/+	ABS/ABS	+/+	+/+	+/+
OAEs	+/+	+/+	+/+	+/+	ABS/ABS	+/+	+/ABS	+/+	+/+	+/+
Gap Det (ms)	5.4	3.8	0.7	6.0	5.0	3.4	11.0	5.6	7.0	6.0
**ABRs**	R/L									
Wave I (ms)	1.55/1.52	1.57/1.67	1.49/1.53	1.49/1.50	ABS/ABS	1.65/2.02	ABS/ABS	ABS/ABS	ABS/ABS	ABS/ABS
Wave III (ms)	4.13/**4.47**	4.01/4.04	4.17/4.01	3.58/3.54	**4.67**/**4.37**	**4.23**/**4.34**	ABS/ABS	ABS/ABS	ABS/ABS	ABS/ABS
Wave V (ms)	6.18/**6.63**	5.81/5.30	5.54/5.33	5.53/5.45	**6.27**/6.08	5.47/**6.26**	**7.08/**ABS	ABS/**7.03**	ABS/ABS	ABS/ABS
IP I–III (ms)	2.58/**2.95**	2.44/2.37	**2.68**/2.48	2.09/2.04	-/-	2.58/2.32		-/-	-/-	-/-
IP III–V (ms)	2.05/2.16	1.80/1.26	1.37/1.32	1.95/1.91	1.60**/**1.71	1.24/1.92		-/-	-/-	-/-


*R/L, right ear/left ear; N, normal; PTA, pure tone average (average thresholds at 0.5, 1, 2, and 4 kHz); LF, low frequencies (average thresholds at 0.5, 1, and 2 kHz); HF, high frequencies (average thresholds at 4, 6, and 8 kHz); ABS, absent; OAEs, otoacoustic emissions; Gap Det., Gap Detection; ABRs, auditory brainstem responses; IP, inter-peak interval. Data are reported in bold when falling beyond the upper limit of the normal range measured for healthy controls in our laboratory. ^∗^Measured ipsilaterally to the stimulated ear.*

In all subjects the onset of vision problems occurred in childhood or the late teens. The auditory symptoms appeared years later as subtle difficulties in understanding speech in noisy environments, except for subject #4, who presented auditory problems as the first symptom. At the time of evaluation, all subjects complained of difficulty in understanding speech in the presence of background noise; four patients also complained of impaired speech perception in quiet environments. Four patients reported tinnitus, whereas one complained of vertigo.

Subjects underwent audiological assessment including pure tone and speech audiometry, speech perception measures and OAEs and ABRs recordings, all performed during the same session. They were submitted to ECochG recording except for subjects #8 and #9.

This study was carried out in accordance with the recommendations of the local institutional ethical committee. All subjects gave written informed consent in accordance with the Declaration of Helsinki.

### Audiological Studies

#### Audiometry

We tested hearing thresholds at frequencies from 250 to 8000 Hz (Grason-Stadler GSI 61 audiometer) in a sound-attenuating room. The degree of hearing impairment was defined by the pure tone average (PTA) threshold levels at 0.5, 1, 2, and 4 kHz, and was classified as mild (PTA 21–40 dB HL), moderate (PTA 41–70 dB HL), severe (PTA 71–95 dB HL), and profound (PTA > 95 dB HL) (study group on terminology, definition and hearing assessment, 1996) (Mazzoli et al., 2003).

#### Speech Audiometry

Articulation gain curves were obtained using disyllabic, phonetically balanced words from an Italian wordlist for adults (Bocca and Pellegrini, 1950). Ten words were presented for each stimulus intensity. At each level, scoring was based on the percentage of words correctly repeated by the subject.

#### Speech Perception Tests

Speech perception tests were performed in a sound-attenuated room. Speech stimuli were presented in the free field through one loudspeaker placed one meter away from the front of the subject’s head. Competing background noise was presented from two additional loudspeakers placed laterally at an angle of 90° on each side of the subject’s head at one meter. Competing noise used was continuous speech noise (Interacoustics AD 229E) calibrated in the free field by means of a Brüel and Kjaer 4165 microphone (mounted on the 800 B Larson-Davis sound level meter) placed in a position corresponding to the subject’s head. Speech material comprised digital anechoic recordings of a native Italian female speaker. The speech stimulus intensity was kept at 70 dB (A). Both stimuli and noise files were stored on computer for transfer directly to the loudspeakers. Speech and noise files were normalized for output intensity levels at the signal-to-noise ratio (*S*/*N*) of 0 dB.

Tests were administered at 70 dB(A) in quiet, and in the presence of competing noise presented at three signal-to-noise (*S*/*N*) ratios (+10, +5, 0). In the word recognition test, subjects were presented with one of four randomly chosen lists of disyllabic words, each list consisting of 25 items. The speech material was obtained from the protocol of patient candidacy for cochlear implantation for the Italian language (Quaranta et al., 1996). Subjects were requested to respond by repeating the words they heard.

Consonant identification was evaluated by presenting consonant confusion matrices compiled from two presentations, each comprising the 16 consonants, b d f g k l m n p r s t v z j t ∫ presented in an “a-consonant-a” context (chance 6%). Subjects were asked to respond by repeating the speech signal perceived.

#### Gap Detection

We used the procedures reported in Zeng et al. (2005). Briefly, tests were performed in free field at an intensity of 30 dB above threshold (with respect to the PTA value calculated for the better ear). Gap detection was evaluated by inserting a silent interval in the center of broadband white noise (20–14,000 Hz). The stimulus paradigm consisted of a three-interval, three-alternative, forced-choice, two-down and one-up procedure to fulfill the 70.7% correct response criterion. White noise was calibrated in the free field by means of a Brüel and Kjaer 4165 microphone (mounted on the 800 B Larson-Davis sound level meter).

Data collected from patients were compared to the corresponding values obtained from 16 normally hearing subjects (age range 18–51 years).

#### Middle Ear Muscle Reflex Testing

Acoustic reflex thresholds were measured ipsilaterally and contralaterally to the stimulated ear (Grason-Stadler GSI TympStar impedance audiometer). They were considered absent when no response was found at intensities higher than 110 dB HL.

Peak latencies were measured on the averaged waveforms obtained in response to tone stimuli of 300 ms duration and 18 ms rise/fall- time which were presented at an intensity of 10 dB above threshold at frequencies of 0.5, 1, 2, and 4 kHz. Five stimuli were averaged for each waveform. Peak latency was measured at the maximum negative deflection from baseline relative to the response onset defined at the time when the immittance signal deviated from the pre-stimulus baseline (Gelfand, 2002).

Data collected from patients were compared to the corresponding values obtained from 16 normally hearing subjects (age range 18–51 years).

#### Distortion Product Otoacoustic Emissions (DPOAEs)

Distortion product otoacoustic emissions (DPOAEs) were obtained using the ILO-92 OAE system. Primary tones were presented at 70 dB SPL and the f2/f1 ratio was kept at 1.21. The frequency was increased in 1/4 octave steps from 708 to 6299 Hz. Four spectral averages were summed for each stimulus condition.

### Electrophysiological Studies

#### Auditory Brainstem Responses (ABRs)

Potentials were recorded from scalp electrodes (vertex to mastoid ipsilateral to the stimulated ear) in response to 2,000 trials of alternating polarity clicks presented monaurally (TDH-50 transducer earphone) at a maximum intensity of 125 dB peak equivalent (p.e.) SPL (corresponding to 90 dB nHL, referred to the psychoacoustical threshold of normally hearing subjects). The filter settings of the amplifier were set between 5 and 4,000 Hz.

#### Electrocochleography (ECochG)

Electrocochleography recording was carried out in 8 out of 10 patients who had signed an informed consent form. ECochG protocol was assessed by the regional body for quality control of clinical and therapeutic procedures (CCHSA, Veneto Region 2007–2010).

Under local anesthesia (lidocaine) a sterile stainless steel needle electrode (0.7 mm), insulated except for the tip, was passed through the tympanic membrane and placed on the promontory wall with the aid of an operating microscope. Stimuli consisted of 0.1 ms rarefaction and condensation clicks, delivered separately in the free field by means of two high frequency drivers (Electro-Voice DH1A/2MT 16 Ω) mounted on a single polyurethane horn (Electro-Voice HP420) at a maximum intensity of 120 dB peak equivalent (p.e.) SPL (corresponding to 90 dB nHL relative to the psychoacoustic threshold of normally hearing subjects). The stimulus was calibrated in the free field by means of a Brüel and Kjaer 4165 microphone (mounted on an 800 B Larson-Davis sound level meter) placed at 1 m from the base of the polyurethane horn, which corresponded to the distance of the patient’s ear from the horn. The procedure of comparing the peak-to-peak amplitude of the click to the peak-to-peak amplitude of a 2 kHz tone was utilized to calibrate the click level (p.e. SPL) (Santarelli et al., 2008).

Condensation and rarefaction clicks were delivered separately for seven intensity levels from 120 to 60 p.e. SPL. The stimulus paradigm consisted of an initial click, followed 15 ms later by ten clicks with an inter-stimulus interval of 2.9 ms, and the sequence was repeated every 191 ms (Santarelli et al., 2008; Santarelli and Arslan, 2013).

The potentials were differentially amplified (50,000 times), filtered (5–8,000 Hz) and digitized (25 μs) for averaging (500 trials). The procedure of averaging the responses evoked separately by condensation and rarefaction clicks was applied to cancel the CM and extract the CAP with the superimposed SP. The resulting curve was subtracted from the potential evoked by condensation clicks to obtain the CM (Eggermont, 1976; Santarelli et al., 2008). Since CM attenuation was often incomplete at high stimulus intensity and CM spectral energy was at maximum between 1,500 and 3,000 Hz, a low pass digital filter (12 dB/octave, cutoff frequency 2,000 Hz) was used to attenuate the residual CM, where needed (Santarelli et al., 2008).

After canceling the CM, the ECochG waveform begins with the receptor SP, which appears as an initial negative deflection arising from baseline and preceding the neural CAP (Eggermont, 1976; Santarelli and Arslan, 2013, 2015). Latency was defined relative to CM onset in milliseconds (ms). Amplitude was computed relative to the period 1 ms before CM onset in microvolt (μV). We defined latency and amplitude of SP at the initial negative deflection arising from baseline while CAP peak amplitude was measured at maximum negative potential (with respect to baseline).

Cochlear potentials recorded from patients were compared to the ECochG data previously collected from 20 children (age range 3.5–6.5 years) tested for presumed cochlear deafness, but who proved to have normal cochlear function (Santarelli et al., 2008; Santarelli and Arslan, 2013, 2015). Although the controls were considerably younger than OPA8 subjects, the age difference cannot be considered a major limitation, since both amplitude and latency of the CAP peak recorded in children of the control group in response to click stimulation at several intensities were comparable to the corresponding values reported in other studies for normally hearing adults (Eggermont, 1976; Noguchi et al., 1999; Schoonhoven, 2007). This is in line with our knowledge of the timing of developmental maturation processes in the cochlea and auditory nerve. Indeed, the latency of ABR wave I, which reflects the synchronous activation of auditory nerve fibers, is comparable to adult values by 1–2 years of life (Eggermont et al., 1991).

### Statistical Analysis

Analysis of variance (ANOVA) for repeated measures was carried out to analyze ECochG and acoustic reflex measures. Separate two-factor ANOVAs with factors of group and stimulus intensity were used to evaluate latency, amplitude and duration measures of ECochG potentials. Factors of group and tone frequency (0.5, 1, 2, and 4 kHz) were used to evaluate acoustic reflex thresholds and peak latencies.

The level of significance was *P* < 0.05.

Values contained in figures indicate mean ± standard error.

## Results

### Hearing Thresholds

Hearing thresholds were within normal limits in 4 subjects and appeared only mildly elevated in five (Table 2). One subject (#9) had normal hearing thresholds in one ear and mild hearing loss in the other ear. In the hearing-impaired patients hearing loss mostly involved the high frequencies.

Subjects were pooled into two groups based on their PTA values. The first group comprised patients with normal hearing thresholds bilaterally (PTA 0–15 dB; *n* = 4), whereas subjects with mild hearing loss in one ear at least (PTA 20-34 dB; *n* = 6) formed the second group.

### Speech Audiometry

**FIGURE 1:**
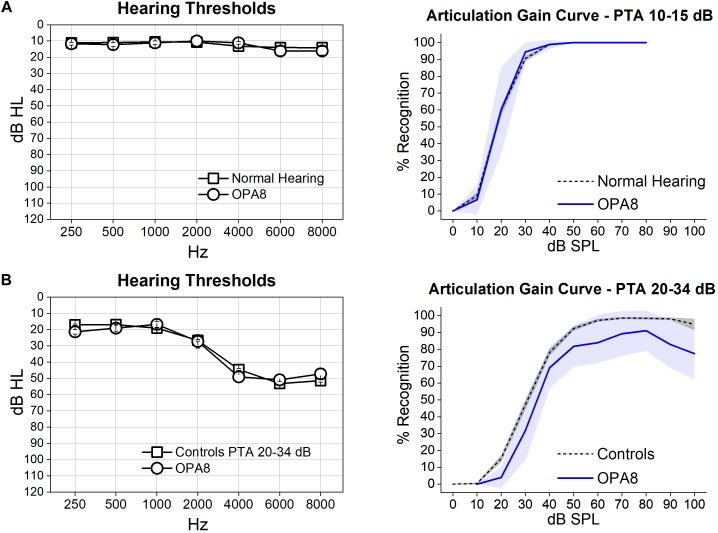
Articulation gain curves from patients with *OPA8*-related DOA are reported for two groups of subjects, one showing normal thresholds **(A)** and the other with mild hearing loss **(B)**. On the left, means and standard errors of hearing thresholds are superimposed on the corresponding values collected from two control age-matched groups with corresponding PTA values. On the right, the mean articulation functions (blue lines) with 95% confidence limits (shadowed areas) obtained from each group of *OPA8* patients have been superimposed on the corresponding mean articulation functions (dashed lines) calculated for controls. Speech intelligibility was within normal limits in the group of *OPA8* patients with normal hearing thresholds, whereas a decrease of reception scores compared to controls was observed in the *OPA8* group with mild hearing loss.

Articulation-gain curves were obtained bilaterally from all subjects. Figure 1 shows the mean hearing thresholds (left side) and the mean articulation-gain curves (right side) obtained from each group of OPA8 patients, compared to the mean functions obtained at corresponding PTA values from two large samples of subjects of comparable age who have undergone audiometric evaluation over the last 10 years at our Department. The two control groups included normally hearing individuals (384 ears, age range 18–47 years) and patients with cochlear hearing loss (575 ears, range 55–69 years), respectively. Subject #9 was included in both classes as she had asymmetrical hearing thresholds.

The mean articulation gain function calculated for the OPA8 ears with normal hearing thresholds (Figure 1A) closely followed the corresponding curve obtained from healthy controls. In contrast, the group of OPA8 patients with mild hearing loss (Figure 1B) showed lower scores compared to the hearing-impaired controls. These findings indicate that the decrease in speech intelligibility in the group of the hearing impaired OPA8 patients cannot be attributed solely to the increase of hearing threshold, as is the case for subjects with cochlear hearing loss.

### Speech Perception

**FIGURE 2:**
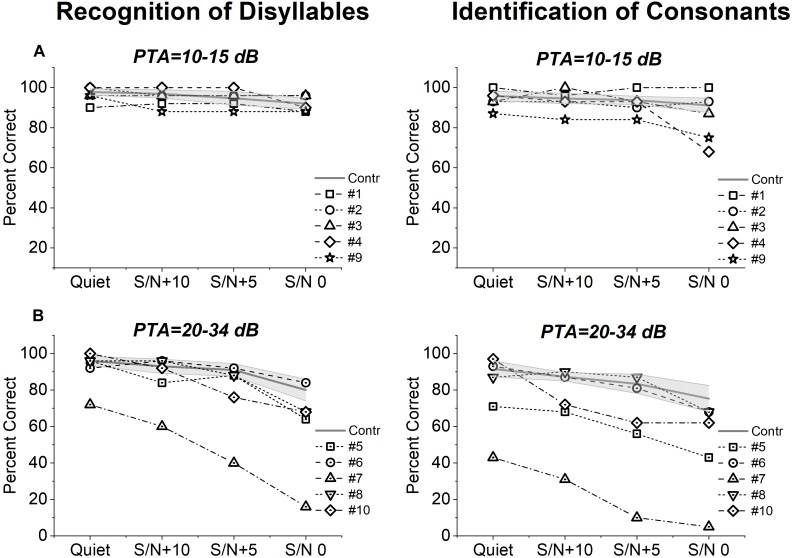
Speech perception scores from patients with *OPA8*-related DOA. Individual scores collected on open-set disyllable recognition and consonant identification tests performed in quiet and in the presence of competing speech noise at three *S*/*N* ratios (+10, +5, 0) are shown for *OPA8* subjects with normal hearing thresholds **(A)** or mild hearing loss **(B)**, superimposed on the mean values with 95% confidence limits (shadowed areas) calculated for two control age-matched groups at corresponding PTA values.

Figure 2 shows individual scores on the recognition of disyllables and identification of consonants obtained from each group of OPA8 patients in a quiet environment and in the presence of background noise, compared with the corresponding mean values measured for two control age-matched groups, one of 14 normally hearing individuals (age range 18–49 years), and the other comprising 10 patients with mild cochlear hearing loss (range 51–69 years). Patient #9 was included in the group of patients with PTA 10–15 dB according to the hearing threshold of the better ear as speech stimuli were presented in the free field.

Speech perception scores measured for OPA8 patients with normal hearing thresholds (Figure 2A) were within the range calculated for healthy controls in three subjects. Of the remaining two patients, subject #4 showed reduced scores on the consonant recognition test in the presence of high level of noise and subject #9 performed worse than controls at all levels of noise particularly on the consonant recognition test. In the OPA8 group with mild hearing loss (Figure 2B), three subjects (subjects #5, #7, #10) performed worse than controls on both tests and particularly on the consonant identification test in the presence of noise, possibly due to the involvement in a more difficult perceptual task. Two subjects performed worse than controls also in quiet (subjects #5, #7). In this group scores were remarkably lower in comparison with OPA8 patients with normal hearing thresholds.

### Gap Detection

Individual values collected on gap detection test are reported in Table 2. In all but one subject (#7), gap detection values were comparable to the scores collected from the group of normally hearing controls (normal gap detection range 2.3–7.5 ms).

### Middle Ear Muscle Acoustic Reflexes

Middle ear muscle reflexes were detected in all but one patient (#5) (Table 2).

**FIGURE 3:**
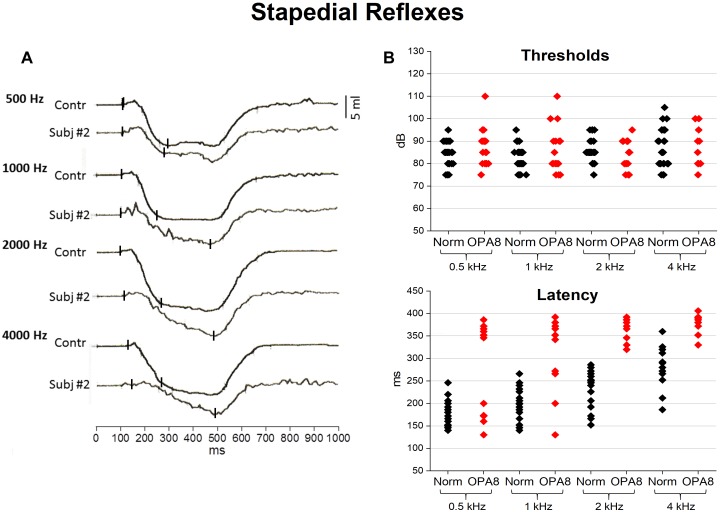
Acoustic middle ear reflexes from *OPA8* patients and normally hearing controls. On the left **(A)** recordings obtained at stimulus frequencies of 500, 1,000, 2,000, and 4,000 Hz at suprathreshold intensity (10 dB above threshold) are shown for one normally hearing individual and one representative OPA8 patient (#2). On the right **(B)**, individual values of reflex threshold and peak latency are graphed for both OPA8 patients and normally hearing controls at stimulus frequencies from 500 to 4,000 Hz. Peak latencies appear remarkably prolonged in OPA8 patients compared to controls. Small vertical bars refer to onset and peak response latency.

In Figure 3A the acoustic reflexes obtained in one representative OPA8 subject (#2) at stimulus frequencies of 500, 1,000, 2,000, and 4,000 Hz at suprathreshold intensity (10 dB above threshold) are compared with the corresponding waveforms recorded from one normally hearing control. In the OPA8 patient, the initial negative deflection slowed down, so that the peak latency was delayed compared to the control.

Individual thresholds and peak latencies are compared for OPA8 patients and one control group of normally hearing individuals in Figure 3B. No significant differences were found in reflex thresholds (ANOVA; *F* = 0.09, *P* = 0.761544), whereas peak latencies were significantly delayed in OPA8 subjects compared to controls (ANOVA; *F* = 45.33, *P* < 0.001).

### Distortion Product Otoacoustic Emissions (DPOAEs)

Distortion product otoacoustic emissions were recorded from all but one patient (#5) (Table 2). The responses were detected bilaterally, except for subject #7, who showed OAEs only in one ear.

### Auditory Brainstem Responses (ABRs)

**FIGURE 4:**
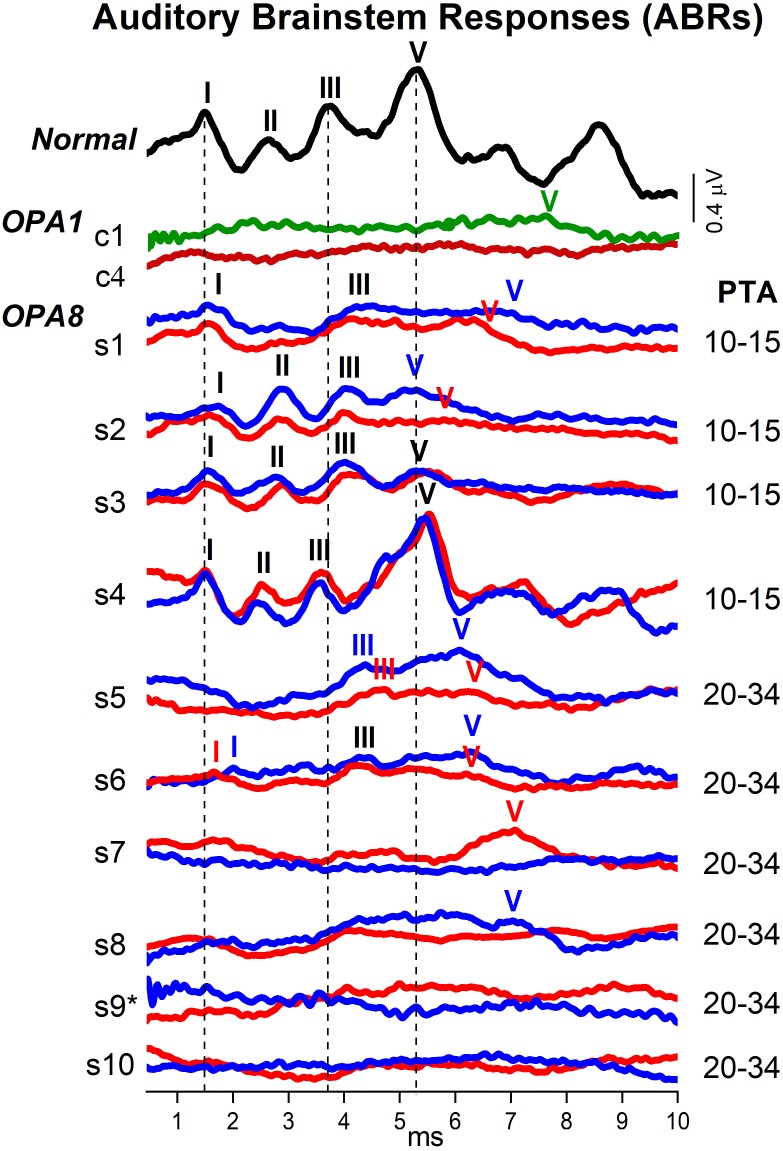
Auditory brainstem responses (ABRs) from patients with OPA8-related DOA. Brainstem potentials recorded at 90 dB nHL are shown for individual OPA8 patients (s1–s10), one normally hearing control and two subjects carrying missense mutations in the *OPA1* gene (c1,c4). Compared to the healthy control, ABRs were markedly attenuated in the *OPA8* group with normal hearing thresholds (PTA 10–15 dB), except for subject #4 who showed normal brainstem potentials. Wave I was detected in all ears with normal latency, whereas Waves III and V were prolonged in almost all ears. ABR abnormalities were much more pronounced in the group of *OPA8* patients with mild hearing loss, spanning from delay of individual ABR waves to absent brainstem potentials, thus resembling the ABR waveforms recorded from patients with OPA1-related DOA. PTA refers to the pure tone average threshold level at 500, 1,000, 2,000, and 4,000 Hz; the potentials collected from the right and left ear are indicated, respectively, by red and blue lines; the vertical dashed lines refer to normal ABR waves peak latency. The asterisk is to indicate that subject #9 had normal hearing threshold in the left ear.

In Figure 4 the individual ABR recordings obtained from all OPA8 subjects are compared to the ABR waveforms collected from one healthy control and from two adult patients carrying a missense mutation in the *OPA1* gene. Peak latency values of individual ABR waves are reported in Table 2.

Overall, ABR amplitudes were attenuated in all OPA8 subjects in comparison with the normally hearing control except for subject #4, who showed normal ABRs. Nevertheless, brainstem potentials showed remarkable differences between OPA8 subjects with normal hearing thresholds and those with mild hearing loss. Compared with the healthy control, the ABR waveforms from the OPA8 group with normal thresholds (PTA 10–15 dB) were markedly attenuated, but the response morphology was preserved. Indeed, Wave I was detected in all ears with normal latency; Waves III and V were identified in all subjects, although with prolonged latency in some ears (bold characters in Table 2). Moreover, in two subjects (#1, #3) the inter-peaks I–III latency in one ear was beyond the upper limit of the normal range measured for healthy controls in our laboratory (2.60 ms) (bold characters in Table 2).

Auditory brainstem response alterations were much more pronounced in the OPA8 group with mild hearing loss (PTA 20–34 dB). Indeed, ABR recordings showed no response in two patients, a remarkably delayed Wave V in two other subjects, while in the remaining two patients Waves III and V were recorded with prolonged latency. Wave I was detected only in one subject in this group (#6). Overall, ABR recordings resembled the brainstem potentials obtained in OPA1 patients, who displayed severe ABR abnormalities despite the relative preservation of hearing thresholds (Santarelli et al., 2015).

**FIGURE 5:**
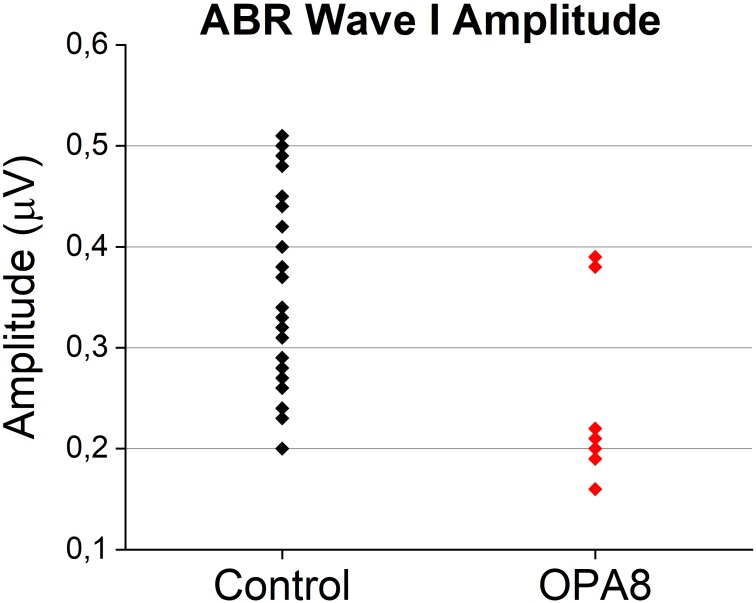
Individual Wave I amplitudes from OPA8 patients showing normal hearing thresholds and a group of normally hearing subjects (*n* = 14). Wave I amplitude was significantly attenuated in OPA8 patients compared to healthy controls.

Since reduction of suprathreshold Wave I amplitude has been proposed as a measure of cochlear input decrease in auditory synaptopathy for both mice and humans (Liberman and Kujawa, 2017), we compared the individual Wave I amplitudes measured at 90 dB nHL for OPA8 patients with normal hearing thresholds with the corresponding values collected from a group of 14 normally hearing subjects (age range 20–57 years) (Figure 5). Wave I amplitude was significantly reduced in the OPA8 group compared to the healthy controls (*t*-test; *t* = 3.09612, *P* < 0.01).

### Electrocochleography (ECochG)

**FIGURE 6:**
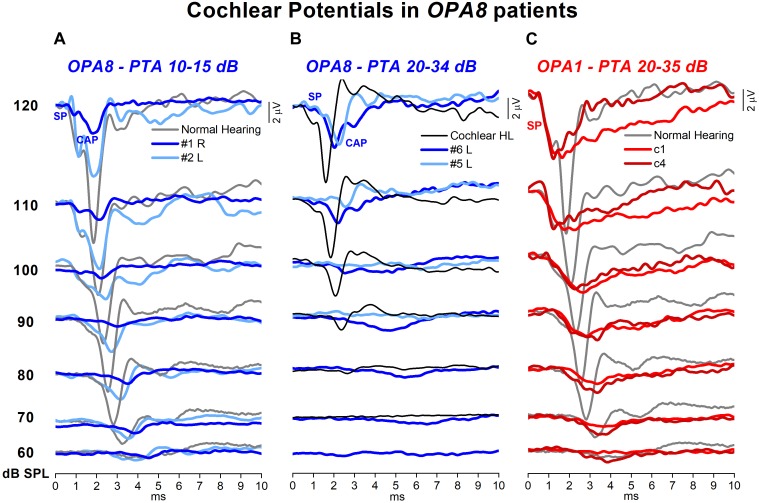
Cochlear potentials recorded from patients with *OPA8*-related DOA. The ECochG responses to clicks at stimulus intensity from 120 to 60 dB p.e. SPL recorded in four representative *OPA8* patients, two with normal hearing thresholds **(A)** and two with mild hearing loss **(B)**, are superimposed, respectively, on the ECochG potentials recorded from two control subjects, one with normal hearing and the other affected by cochlear hearing loss. Compared to controls, cochlear potentials recorded in *OPA8* patients were attenuated in amplitude, delayed in peak latency and prolonged in duration, these changes being more pronounced in the two OPA8 patients with mild hearing loss. In subject #2 (right ear), however, SP amplitude and latency were of control values. In the right panel **(C)**, the ECochG waveforms collected from two patients with AN associated with missense mutations in the *OPA1* gene are shown for comparison. In this and in the subsequent figures time “0” refers to CM onset.

Electrocochleography waveforms obtained after CM cancelation showed remarkable differences in comparison with normally hearing individuals and subjects with cochlear hearing loss. In Figure 6 the ECochG waveforms recorded at stimulus intensities from 120 to 60 dB p.e. SPL in four representative OPA8 patients – two with normal hearing thresholds (A) and two with mild hearing loss (B) – are superimposed, respectively, on the ECochG potentials recorded from two control subjects: a normally hearing individual and a subject with cochlear deafness with comparable hearing thresholds. In addition, recordings collected in two patients with auditory neuropathy due to mutations in the *OPA1* gene are displayed in the right panel. In the healthy control (Figure 6A), the response begins with the receptor summating potential (SP), which is believed to derive from IHCs activation (Durrant et al., 1998). This is followed by the CAP, originating from the synchronous activation of auditory nerve fibers innervating the basal portion of the cochlea (Eggermont, 1976). Decreasing the stimulus level results in a gradual latency increase and amplitude reduction of both SP and CAP peaks. The duration of the SP-CAP complex, as measured from initial negative deflection to return to baseline, is relatively constant at suprathreshold intensities but broadens at low stimulus level. The ECochG waveforms obtained from the subject with cochlear deafness (Figure 6B) show comparable peak latencies and duration with respect to the normally hearing control; however, the amplitude of both SP and CAP is remarkably smaller.

The ECochG waveforms recorded from OPA8 patients began with the SP potential, whose amplitude appeared markedly reduced compared to the controls for OPA8 subjects with normal hearing thresholds (Figure 6A) as well as for those showing mild hearing loss (Figure 6B). SP potential was followed by synchronous CAP, which displayed a smaller amplitude and a delayed peak latency compared to controls for both OPA8 groups. The attenuation in amplitude of ECochG potentials and the delay of SP and CAP peak latency were less pronounced in the group of OPA8 patients with normal hearing thresholds. Indeed, subject #4 had normal ECochG responses (not shown), while the ECochG waveforms recorded from the right ear in subject #2 (Figure 6A) showed reduced CAP amplitude and increased peak latency compared to the control; however, both SP amplitude and latency were within the control range. These findings support the hypothesis that in the youngest members of the OPA8 family, synaptic damage was far less advanced compared to elderly patients.

The pattern of ECochG responses showed profound differences in comparison to patients harboring missense mutations in the *OPA1* gene, as in the latter the synchronous CAP was replaced by a prolonged low-amplitude negative potential following an SP with a normal amplitude (Figure 6C).

**FIGURE 7:**
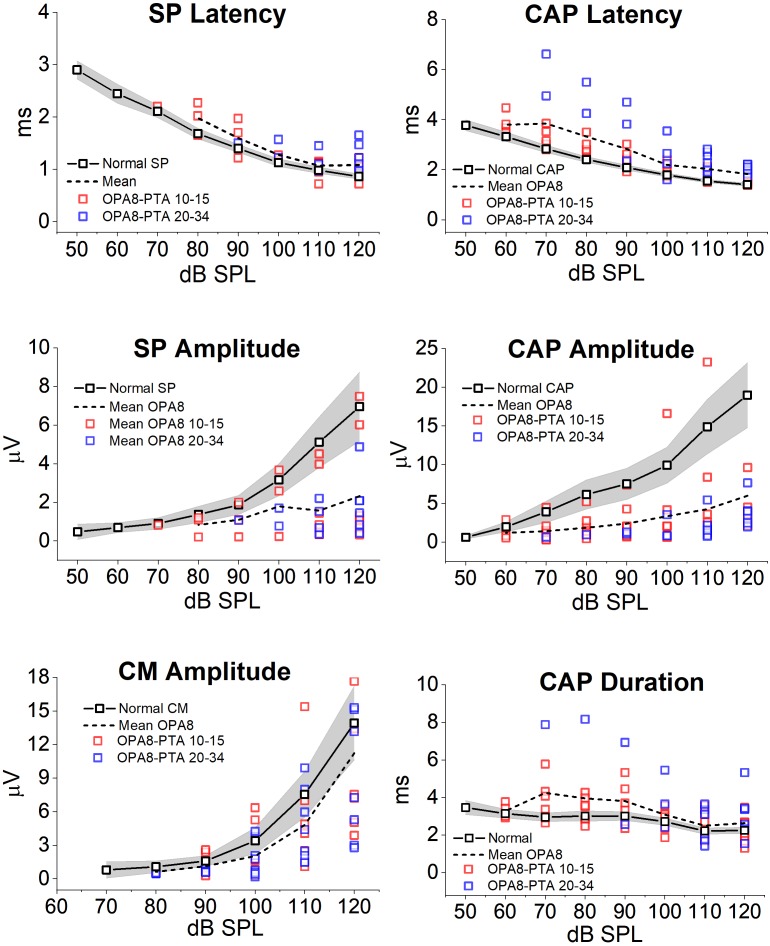
Amplitude, latency and duration of ECochG potentials recorded from OPA8 patients. Individual (colored squares) and mean values (dashed line) of amplitude, latency and duration of ECochG potentials obtained in OPA8 patients are plotted as a function of signal intensity superimposed on the corresponding mean values (continuous line, black open squares) with 95% confidence limits (shadowed areas) calculated for normally hearing controls. Compared to controls, the SP and CAP recorded in OPA8 subjects were decreased in amplitude and increased in peak latency and duration, whereas no differences were found in CM amplitudes. Differences between OPA8 patients and controls were more pronounced for subjects with mild deafness (blue squares) compared to those with normal hearing thresholds (red squares).

Mean and individual values of amplitude, latency and duration of ECochG potentials recorded in OPA8 patients are plotted as a function of signal intensity in Figure 7, superimposed on the corresponding mean values with 95% confidence limits (shadowed areas) calculated for normally hearing controls. No significant differences were found in CM amplitude (ANOVA; *F* = 1.31, *P* = 0.252) in OPA8 patients compared to controls, whereas SP was significantly reduced in amplitude (ANOVA; *F* = 4.60, *P* = 0.041) and delayed in peak latency (ANOVA; *F* = 4.48, *P* = 0.044). Compared to normally hearing controls, the CAP recorded in OPA8 subjects was significantly decreased in amplitude (ANOVA; *F* = 7.25, *P* = 0.012) and increased in both peak latency (ANOVA; *F* = 19.17, *P* < 0.001) and duration (ANOVA; *F* = 10.14, *P* < 0.05) (Figure 6). Overall, SP and CAP potentials recorded in OPA8 patients with mild hearing loss had higher thresholds, smaller amplitudes, delayed peak latencies and increased durations in comparison with the group of OPA8 subjects with normal thresholds.

**FIGURE 8:**
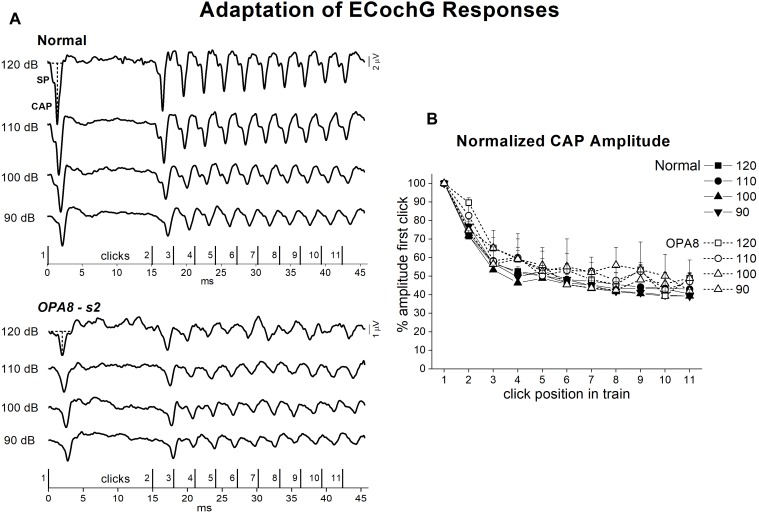
Adaptation of ECochG potentials in *OPA8*-related DOA. ECochG waveforms recorded at stimulation intensities from 120 to 90 dB p.e. SPL in one normally hearing control and in one OPA8 patient (s2) in response to the stimulus sequence reported at the bottom are displayed in the left panel **(A)**. On the right **(B)**, the mean and standard errors of normalized CAP amplitudes are reported as a function of click position in the stimulus sequence for both controls and OPA8 patients. The size of attenuation of cochlear potentials during adaptation calculated for OPA8 subjects was within the range of the CAP attenuation calculated for controls. The horizontal and vertical dashed lines indicate baseline and CAP peak, respectively.

The effects of adaptation on the cochlear potentials were studied by using a stimulation paradigm at high rates (Eggermont and Odenthal, 1974; Santarelli and Arslan, 2013). Figure 8A shows the ECochG waveforms obtained at several intensities in a normally hearing control and in one representative OPA8 patient in response to the sequence of click stimuli reported at the bottom. In the normal control, CAP amplitude showed a marked attenuation from the first (#1) to the second (#2) click in the sequence (which corresponded to the first click in the high-rate train), then a further drop was apparent during the sustained train sequence. A similar behavior was observed in the OPA8 patient, although SP and CAP amplitudes were much smaller. In Figure 8B the means and standard errors of ECochG response amplitudes calculated for both normally hearing controls and OPA8 patients are reported as a function of click position in the stimulus sequence at intensities from 120 to 90 dB p.e. SPL. Amplitudes were normalized to the first click in the sequence. The amount of amplitude reduction obtained in OPA8 patients at each stimulus level proved to be close to that calculated for controls, consistent with a normal adaptation pattern of auditory nerve fibers.

## Discussion

Our study demonstrates that the hearing dysfunction in OPA8 patients is underlain by hidden auditory neuropathy due to degeneration of synaptic contacts between hair cells and auditory nerve fibers. This results in abnormalities of the evoked auditory nerve (SP, CAP) and ABRs potentials, which constitutes the first sign of the disease, whereas hearing thresholds and speech perception appear to be largely unaffected at an early stage. As the disease progresses with age, hearing thresholds deteriorate, speech perception difficulties arise and both CAP and ABRs become further impaired.

We have previously characterized the hearing dysfunction affecting patients with DOA due to missense mutations in the *OPA1* gene (Santarelli et al., 2015), who display impaired speech perception, severe abnormalities of ABRs and presence of OAEs consistent with auditory neuropathy. Although the optic neuropathy found in the OPA8 family seems indistinguishable from that described in the OPA1 disease (Carelli et al., 2011), the hearing dysfunction appears profoundly different. First, in OPA8 patients the onset of hearing symptoms is delayed compared to OPA1 subjects. Moreover, the hearing impairment mainly consists of difficulties in understanding speech in the presence of noise, whereas in OPA1 patients the speech perception is severely impaired also in quiet environments. Most importantly, the combination of audiological and electrophysiological findings points to different pathophysiological mechanisms and sites of lesion.

Although complaining of subtle difficulties in understanding speech such as great effort involved in listening in the presence of noise, the youngest members of the OPA8 family showed normal hearing thresholds and speech perception scores. Interestingly, the only OPA8 patient with normal hearing thresholds who showed a remarkably reduced performance on speech perception tests, was 69 years old (Table 2), and had normal hearing thresholds in one ear and mild hearing loss in the other ear. Looking at the oldest patients, who showed mild deafness bilaterally, the comparison of the mean intelligibility scores with those obtained from a group of subjects with cochlear deafness of the same age, revealed lower performances, thus indicating that their difficulties in speech understanding cannot be attributed solely to the hearing threshold elevation or to presbycusis. Differences between OPA8 patients and controls appeared even more striking in speech perception tests, particularly when administered in the presence of competing noise.

Otoacoustic emissions and CM amplitudes were normal in OPA8 patients, consistent with preservation of OHCs function, whereas ABRs were abnormal in all but one subject. Specifically, in the youngest patients with normal thresholds, ABR morphology was preserved with detection of the main ABR waves; however, the amplitude of ABR components was markedly attenuated. Recent studies have reported that reduction of Wave I amplitude constitutes the first sign of cochlear damage in animals exposed to noise or in aging mice showing preserved cochlear receptors (Liberman and Kujawa, 2017). This finding has been attributed to diffuse loss of the IHCs synapses following primary degeneration of unmyelinated neural terminals. An attenuation of Wave I amplitude has also been found in humans suffering from tinnitus associated with preservation of hearing threshold (Schaette and McAlpine, 2011). The group of OPA8 patients with normal hearing thresholds also showed a significant reduction of Wave I amplitude compared to healthy controls, consistent with degeneration of IHCs synapses. Moreover, two subjects also showed prolongation of the I–III interval latency. This might indicate a slowing of signal conduction in the cochlear nerve possibly resulting from damage to auditory nerve fibers. However, the prolongation of the I–III interval latency could also result from the decrease of the cochlear output related to synaptopathy (Don and Kwong, 2002). ABR abnormalities were much more pronounced in OPA8 patients with mild deafness, who showed profound alterations of ABR morphology despite mild elevation of hearing thresholds.

Acoustic reflexes to ipsilateral stimulation were detected in all but one OPA8 patient. However, the reflex morphology in response to 300 ms-tone stimuli was abnormal, in that the first rapid deflection from baseline was almost completely abolished with consequent delay of response peak latency, which appeared significantly higher in comparison to healthy controls. Recently, Valero et al. (2018) have shown that acoustic reflexes are attenuated in mice affected by cochlear synaptopathy induced by noise exposure. As proposed for these noise-exposed mice, the slope reduction of acoustic reflexes in OPA8 patients could result from a decrease of rapid low-SR fiber activity, which is known to dominate the afferent limb of the reflex (Kobler et al., 1992). Therefore, together with the reduction of Wave I amplitude, the peak delay of acoustic reflexes constitutes the first sign of AN in OPA8 patients.

ECochG recordings revealed alterations of cochlear potentials in all OPA8 subjects. Both SP and CAP showed a striking reduction in amplitude, consistent with a reduction of cochlear output. This finding accords with the suprathreshold attenuation of ABR Wave I amplitude; however, it constitutes a much more reliable index of cochlear output reduction. A decrease of SP and CAP amplitude is also apparent in cochlear deafness (Santarelli and Arslan, 2013; Santarelli et al., 2016) because of reduced cochlear sensitivity related to loss of the cochlear amplifier. However, differently from patients with cochlear lesions, OPA8 subjects also showed an increase of both CAP peak latency and duration, consistent with a selective reduction of rapid low-SR fibers activity. The attenuation in amplitude of ECochG potentials and the delay of SP and CAP peak latency were more pronounced in the group of patients with mild hearing loss, who were the oldest members of OPA8 family. These patients also showed a remarkable increase of CAP thresholds despite mild hearing threshold elevation. These findings corroborate the hypothesis that in the youngest members of the OPA8 family synaptic damage was far less advanced.

Taken together, speech perception measures and ABR and ECochG findings, may indicate that the youngest members of the OPA8 family show subtle hearing dysfunctions associated with reduction in amplitude and increase in latency of auditory evoked potentials. As the disease progresses over time, auditory perceptions worsen, ABRs are absent or show profound alterations, while CAP shows further attenuation in amplitude and increase in peak latency. Thus, alterations of ABR and ECochG potentials seem to mirror the progressive deterioration of speech perception abilities. Similar changes have been reported by Rance et al. (2012) in two patients with Friedreich ataxia. One of these subjects showed ABR alterations worsening with time, which were mirrored by progressively decreasing spatial listening abilities. In contrast, no changes in ABR potentials were observed during longitudinal assessment in the second patient who retained binaural processing abilities.

ECochG recordings obtained from OPA8 patients showed profound differences in comparison to subjects harboring OPA1 mutations, who showed the typical profile of classical post-synaptic AN. In the latter, the synchronous CAP was replaced by a prolonged low-amplitude negative potential, which is believed to result from activation of damaged neural fibers (Santarelli et al., 2015). In contrast, in OPA8 patients the morphology of the SP-CAP complex was preserved, thus indicating preservation of the dynamics of activation of auditory nerve fibers. This hypothesis is corroborated by the time course of adaptation of CAP potentials during stimulation at high frequency. The amplitude of CAP responses, in fact, decreased during adaptation, and the amount of attenuation closely followed that calculated for normally hearing controls. Wynne et al. (2013) have measured the changes of Wave V amplitude evoked by rapid trains of clicks in patients affected by AN. They found that Wave V was recorded in healthy controls in response to every click of the train sequence, whereas in patients with hereditary neuropathy due to auditory fiber degeneration, Wave V was recorded only in response to the initial click of the stimulus sequence consistent with a neural conduction block. In OPA8 patients, the identification of a well-synchronized CAP in response to every click of the train sequence and the time course of adaptation rule out the hypothesis of a conduction block possibly related to auditory nerve fiber degeneration.

Overall, ECochG recordings from OPA8 family are consistent with the findings obtained by Salvi et al. (2017) in chinchillas after administration of the ototoxic agent carboplatin. Moderate doses of this toxic agent resulted in degeneration of the afferent terminals at the base of IHCs followed by diffuse IHCs loss. Following carboplatin administration an attenuation of both SP and CAP potentials was observed in ECochG recordings, the amount of amplitude reduction being related to the amount of IHCs loss as evaluated histologically. It is noteworthy that the hearing thresholds measured in chinchillas after carboplatin administration were preserved in the presence of small IHCs lesions (<35% of IHCs loss) and were only slightly increased after moderate IHCs loss (40–75%). Thus, we speculate that both the audiological and electrophysiological alterations observed in the two groups of OPA8 patients, young adults and elderly individuals, reflect different amounts of IHCs synaptic degeneration.

Although behavioral hearing thresholds measured in quiet environments in chinchillas were not affected until IHC loss exceeded about 40%, hearing thresholds obtained in the presence of noise were significantly elevated compared to controls in animals with small IHCs lesions. This was explained by admitting that the central auditory system is able to compensate for a small decrease in the cochlear output so that cochlear damage becomes apparent only in challenging conditions. Indeed, Salvi et al. (2017) found that the evoked potentials recorded from the auditory cortex of chinchillas in response to tone-burst stimuli were remarkably enhanced after carboplatin administration. Based on this finding, they hypothesized that the relative preservation of hearing thresholds after administration of low-to-moderate doses of carboplatin results from compensatory mechanisms set in action by the central auditory system, which increases its gain in the attempt to counteract the reduction of cochlear output. The effectiveness of these compensatory effects is likely to be reduced in more challenging conditions such as hearing in the presence of noise, particularly when the percentage of IHCs loss exceeds 50%.

Similar mechanisms might come into play in OPA8 patients. In younger subjects the preservation of both hearing thresholds and speech perception may be achieved by central compensatory mechanisms counteracting the attenuation of the cochlear output, whereas in older subjects both the deterioration of hearing thresholds and the impairment of speech perception could possibly reflect a decreased effectiveness of central compensating mechanisms in counteracting a further decrease of cochlear output. This hypothesis is also supported by the findings reported by Chambers et al. (2016), who observed increased gains in firing rate of auditory cortex neurons in mice after lesioning cochlear nerve synapses through round window application of ouabain. After ouabain administration auditory nerve activity was severely depressed, whereas tone-evoked responses in auditory cortex progressively recovered to firing levels which were even greater than control values. In contrast, spike timing precision and decoding accuracy for pulse trains and speech tokens remained impaired.

**FIGURE 9:**
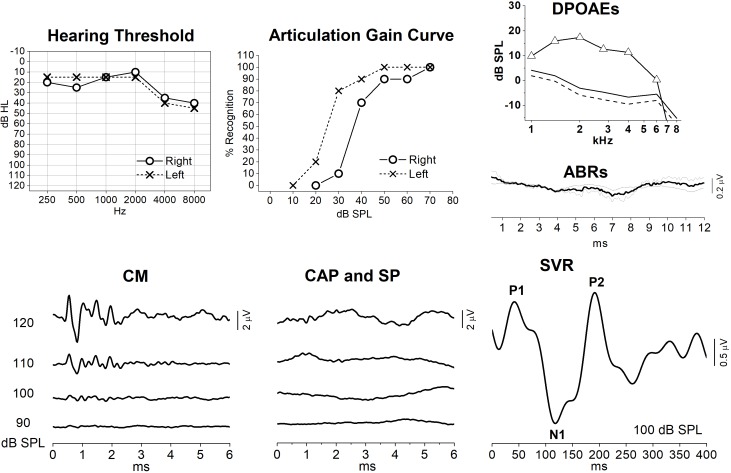
Audiometric and electrophysiological findings from one patient (subj. #10) with OPA8-related DOA. Hearing thresholds and articulation gain curves indicate bilateral mild hearing loss and slight reduction of speech intelligibility in the right ear. OAEs and auditory evoked potentials (ABRs, ECochG and slow vertex potentials, SVR) recordings are reported only for the right ear. DPOAEs were detected with normal amplitude, whereas ABRs were absent. ECochG recordings showed only the CM potential, whereas no SP or CAP responses were detected despite the relative preservation of hearing thresholds. Slow vertex cortical (SVR) potentials were recorded despite the absence of synchronized auditory nerve response.

Interestingly, the data obtained in this animal model resembled the findings we collected from subject #10, who showed mild hearing loss associated with a slight reduction in speech intelligibility (Figure 9) together with decreased speech perception in the presence of noise. In this subject, OAEs were detected bilaterally, whereas ABRs were absent. ECochG recordings showed only the CM potential, whereas no SP or CAP responses were detected despite the relative preservation of hearing thresholds. In contrast, Slow Vertex Potentials (SVR) were clearly recorded in response to click stimuli. Long-latency responses with small amplitude and delayed latency have been obtained in other patients with AN (Starr et al., 1996). This finding was attributed to the relatively longer duration of cortical evoked responses compared to brainstem potentials, the latter requiring a highly synchronous cochlear nerve activity for their generation. Nevertheless, the findings of relatively preserved speech perception in quiet environments together with robust SVR recordings, which were comparable in both amplitude and peak latency to those evoked in normally hearing individuals (Martin et al., 2007), corroborate the hypothesis that an increase in gain of the central auditory system compensates for the cochlear output reduction in OPA8 subject #10.

## Conclusion

We document that the hearing dysfunction affecting OPA8 patients is underlain by hidden AN, possibly resulting from terminal afferent degeneration followed by IHCs loss. The functional alterations consist of abnormalities of both ABR and ECochG potentials together with poor morphology of middle ear acoustic reflexes at an early stage of the disease, whereas mild hearing loss and impairment of speech perception become apparent only at an advanced stage. Central mechanisms are likely to counteract the decrease of peripheral input and guarantee full compensation at an early stage of the disease.

These findings provide a distinctive phenotypic profile of DOA associated with the OPA8 locus as compared with the OPA1-related DOA, which was associated to the classical profile of post-synaptic AN. Elucidation of the molecular pathogenesis will certainly shed further light on the selective auditory terminals and IHCs damage in patients with OPA8-related DOA.

## Author Contributions

RS and VC designed research. RS wrote the manuscript. CLM, MLV, PB, and AM performed experiments and collected data. PS analyzed data. VC provided critical revision and commented on the manuscript at all stages. All authors contributed to this work.

## Conflict of Interest Statement

The authors declare that the research was conducted in the absence of any commercial or financial relationships that could be construed as a potential conflict of interest.
